# Estimated excess acute-care length of stay and extra cost of testing-based versus symptom-based isolation strategies among veterans hospitalized with coronavirus disease 2019 (COVID-19) discharging to a congregate setting

**DOI:** 10.1017/ice.2020.1295

**Published:** 2020-10-26

**Authors:** Chenwei Wu, Susan Glass, Sandra Demars, Luis G. Tulloch-Palomino, Pandora L. Wander

**Affiliations:** 1 Hospital and Specialty Medicine, Veterans Affairs Puget Sound Health Care System, Seattle, Washington; 2 Division of General Internal Medicine, Department of Medicine, University of Washington School of Medicine, Seattle, Washington; 3 Division of Allergy and Infectious Diseases, Department of Medicine, University of Washington School of Medicine, Seattle, Washington

During the coronavirus disease 2019 (COVID-19) pandemic, hospitals worldwide have experienced capacity shortfalls because of high case volumes compounded by prolonged length of stay (LOS).^1^ Although illness severity plays a major role in prolonging hospitalization, asymptomatic or recovered patients could potentially recuperate in congregate settings but may be prevented from discharging due to persistent yet clinically inconsequential viral shedding.^1-7^ Because COVID-19 transmission is unlikely >10 days after symptom onset,^3-5^ the US Centers for Disease Control and Prevention (CDC) in August 2020 recommended symptom-based rather than testing-based isolation measures.^7^ Implications of these recommendations for hospital operations remain unclear. We studied veterans hospitalized with COVID-19 who required discharge to a congregate setting (rehabilitation center, skilled nursing facility, public shelter, or supervised long-term care) to determine the impact of a symptom, rather than testing-based isolation strategy on acute care LOS and cost.

## Methods

We included adults diagnosed with COVID-19 by nasopharyngeal or mid-turbinate specimen reverse-transcriptase polymerase chain reaction assay (RT-PCR) using the Cepheid Xpert Xpress test (Cepheid, Sunnyvale CA) or the Abbott M2000 test (Abbott Laboratories, Abbott Park, IL) between March 2 and June 2, 2020, within the Veterans Affairs Puget Sound Health Care System (VAPSHCS), a large, integrated, federal healthcare network serving western Washington State. Discharge to congregate setting was contingent on 2 consecutive negative RT-PCR tests >24 hours apart.

Trained physician reviewers (C.W., L.G.T., S.G., S.D., and P.L.W.) collected the following data from the electronic health record: hospital admission, discharge, and testing dates. Severe illness and immunocompromised status were defined using CDC criteria (Table 1).^7^ The discharge eligibility date was established retrospectively according to CDC guidance (eg, afebrile, symptomatic improvement, and >10 days from onset or first positive RT-PCR for mild or asymptomatic disease if immunocompetent and >20 days if illness was severe or immunocompromised) or upon resolution of other conditions requiring hospitalization, whichever occurred later.^7^ All charts underwent independent review by a second, blinded investigator; in cases of disagreement, the later discharge eligibility date was used.

**Table 1.** Individual-Level Characteristics, Acute-Care LOS, and Cost Parameters for VA Puget Sound Health Care System Veterans Hospitalized With COVID-19 Between March 2 and June 2, 2020, Needing Discharge to a Congregate Setting^a^

Case	Age, y	Severe Illness^b^	Immuno-compromise^c^	Total LOS, d^d^	Acute-Care LOS, d^d^	Total Acute-Care Cost, USD^d^	Daily Acute- Care Cost, USD^e^	Excess LOS, d^f^	Excess Cost, USD^g^	Prehospital Habitation	Disposition	Principal Diagnosis, ICD-10-CM
1	96	No	No	22	22	$76,368	$3,471	21	$72,897	Long-term care	Rehabilitation (VHA)	U07.1
2	89	Yes	No	32	32	$106,911	$3,341	13	$43,433	Long-term care	Rehabilitation (VHA)	U07.1
3	71	Yes	No	35	35	$148,011	$4,229	8	$33,831	Long-term care	Rehabilitation (Non-VHA)	A41.9
4	76	No	No	45	45	$185,841	$4,130	27	$111,505	Long-term care	Rehabilitation (VHA)	I99.8
5	76	Yes	No	19	17	$60,961	$3,586	2	$7,172	Homeless	Shelter	U07.1
6	74	Yes	No	46	8	$42,676	$5,335	1	$5,335	Private residence	Rehabilitation (VHA)	U07.1
7	71	Yes	No	44	5	$14,991	$2,998	0	$0	Private residence	Rehabilitation (VHA)	A41.89
8	73	Yes	No	29	29	$104,620	$3,608	20	$72,152	Long-term care	Rehabilitation (VHA)	U07.1
9	74	Yes	No	33	33	$121,513	$3,682	20	$73,644	Homeless	Quarantine (VHA)^8^	I13.0
10	100	Yes	No	28	28	Missing	Missing	3	Missing	Long-term care	Long-term care	Missing
11	68	Yes	Yes	38	21	$91,091	$4,338	8	$34,701	Private residence	Rehabilitation (VHA)	U07.1
			**Total**	371	275	$952,983	N/A	123	$454,669			
			*Median*	33	28	$97,856	$3,645	8	$39,067			

Note. COVID-19, coronavirus disease 2019; LOS, length of stay; VHA, Veterans Health Administration; CDC, US Centers for Disease Control and Prevention; ICD-10-CM, diagnostic codes per the *International Classification of Diseases, 10^th^ Edition, Clinical Modification.*

a Refers to rehabilitation center, public shelter, or supervised long-term care setting such as an adult family home.

b Defined by CDC as respiratory rate >30, sea-level oxygen saturation <94% on ambient air or >3% decrease from baseline in patients with chronic hypoxemia, ratio of arterial partial pressure of oxygen to fraction of inspired oxygen <300, or lung infiltrates >50% on chest imaging.

c Defined by CDC as ongoing chemotherapy, untreated human immunodeficiency virus infection with CD4 cell count <200, combined primary immunodeficiency disorder, daily prednisone dose >20 mg/d for >14 d, or <12 mo since hematologic or solid-organ transplant.

d Figures obtained from the VHA Allocation Resource Center.

e Calculated by dividing total acute-care cost by acute-care LOS.

f Calculated by subtracting the date of actual discharge by the date when discharge could have occurred under CDC symptom-based recommendations.

g Calculated by multiplying excess LOS by the daily acute-care cost.

h Patient discharged to dedicated ambulatory quarantine site to await 2 negative test results before final disposition to shelter 8 days later.

Excess acute-care LOS was defined as the difference between the true discharge date and the discharge eligibility date. Excess cost of care was determined by multiplying the “excess” fraction of a patient’s stay by the total acute-care cost reported by the Veterans Health Administration (VHA) Allocation Resource Center (ARC). ARC costs are based on the managerial cost accounting system used widely in VHA cost-effectiveness research, adjusted for administrative overhead and special fees.^8-10^ Emergency department and intensive care costs were excluded. This study was approved by the VAPSHCS Institutional Review Board. The requirement for informed consent was waived.

## Results

Overall, 70 veterans were diagnosed with COVID-19 during the study period and 29 (41.4%) required hospitalization. Among them, 11 (37.9% of hospitalized cases) were discharged to a congregate setting and were included in this analysis. Furthermore, 10 (90.9%) were admitted to VAPSHCS; 1 was admitted to a community hospital and lacked cost information. All were male, with median age of 74 years (range, 68–100). In addition, 9 (81.8%) had severe illness and 1 (9.1%) was immunocompromised due to solid-organ transplantation (Table 1).

Among this cohort, 7 patients (63.6%) were discharged to a VHA-managed rehabilitation unit. One individual originally destined for this setting improved sufficiently to relocate to a VHA ambulatory quarantine site. One patient was discharged to a non-VHA rehabilitation center, 1 was discharged directly to shelter, and 1 was discharged to his prior adult family home (Table 1).

Testing-based isolation practices generated a cumulative 123 excess bed days of care and $454,669 in additional cost. Median excess acute-care LOS was 8 days (range, 0–27 days). Among 10 patients with ARC financial data, median excess cost was $39,067 (range, $0–$111,505) and cost per additional inpatient day was $3,645 (range, $2,998–$5,335). In total, 275 bed days and $952,983 were spent in acute care, of which >40% (123 days and $454,669) could have been avoided using new symptom-based recommendations (Table 1).

## Discussion

In our analysis, postsymptomatic inpatients with COVID-19 needing discharge to a congregate setting remained hospitalized a median 8 days longer and generated nearly $40,000 in additional cost per person under a testing-based rather than symptom-based isolation strategy. Symptom-based precautions could have reduced the total LOS and cost by >40%. These calculations were made using conservative interpretations of the discharge eligibility date, which may result in underestimates. To our knowledge, this is the first analysis of operational consequences for facilities following a testing-based rather than a symptom-based isolation strategy. Our findings suggest nontrivial benefits of the latter, both in LOS reductions as well as cost savings.

Moreover, 8 of the 11 patients (72.3%) discharged to the VHA, rather than privately operated postacute care settings, including 1 to a specialized VHA quarantine site. Had ambulatory quarantine not been available, an additional 8 inpatient days and $26,600 in cost would have accrued prior to achieving testing-based clearance. The existence of VHA-managed rehabilitation units and dedicated quarantine sites demonstrates a degree of operational flexibility that may not be shared by more fragmented community hospitals and their post-acute care partners. These system-level differences raise the possibility that the LOS and cost differential for a testing-based versus symptom-based isolation strategy may be amplified in the private sector.

The strengths of our study include the incorporation of true per-patient costs and chart review performed by trained clinicians blinded to those costs. Limitations include small sample size and limited generalizability.

These preliminary results suggest healthcare administrators and governmental authorities should act quickly to translate symptom-based isolation strategies into practice. They also stress the importance of establishing clinically significant transmission criteria, rather than relying on highly sensitive molecular assays alone, to inform isolation guidelines in future outbreaks.
